# A Facile Strategy for Visualizing and Modulating Droplet-Based Microfluidics

**DOI:** 10.3390/mi10050291

**Published:** 2019-04-29

**Authors:** Zehang Gao, Huo Peng, Minjie Zhu, Lei Wu, Chunping Jia, Hongbo Zhou, Jianlong Zhao

**Affiliations:** 1 State Key Laboratory of Transducer Technology, Shanghai Institute of Microsystem and Information Technology, Chinese Academy of Sciences, Shanghai 200050, China; gaozh@shanghaitech.edu.cn (Z.G.); ph152801@mail.sim.ac.cn (H.P.); 13867619779@163.com (M.Z.); wulei@mail.sim.ac.cn (L.W.); jiachp@mail.sim.ac.cn (C.J.); 2 School of Information Science and Technology, ShanghaiTech University, Shanghai 201210, China; 3 University of Chinese Academy of Sciences, Beijing 100049, China; 4 Department of Chemistry, College of Science, Shanghai University, Shanghai 200444, China

**Keywords:** microfluidics, droplets, high-throughput, modulate

## Abstract

In droplet-based microfluidics, visualizing and modulating of droplets is often prerequisite. In this paper, we report a facile strategy for visualizing and modulating high-throughput droplets in microfluidics. In the strategy, by modulating the sampling frequency of a flash light with the droplet frequency, we are able to map a real high frequency signal to a low frequency signal, which facilitates visualizing and feedback controlling. Meanwhile, because of not needing synchronization signals, the strategy can be directly implemented on any droplet-based microfluidic chips. The only cost of the strategy is an additional signal generator. Moreover, the strategy can catch droplets with frequency up to several kilohertz, which covers the range of most high-throughput droplet-based microfluidics. In this paper, the principle, setup and procedure were introduced. Finally, as a demonstration, the strategy was also implemented in a miniaturized picoinjector in order to monitor and control the injection dosage to droplets. We expect that this facile strategy supplies a low-cost yet effective imaging system that can be easily implemented in miniaturized microfluidic systems or general laboratories.

## 1. Introduction

Microfluidics allows for precise control of flow and the balance between surface tension and viscous forces in multiphase flows, making it possible to generate highly monodisperse droplets [1,2]. Owing to their isolation, high uniformity and throughput, droplets from microfluidic devices have a wide range of applications in biological assays and materials synthesis [3,4,5]. Specifically, droplet microfluidics have been used for digital polymerase chain reaction (PCR) [6], digital ELISA [7], single cell analysis [8], as well as for the synthesis of functional hydrogels and particles [9,10]. In these applications, high-throughput droplets and the associated visualization and modulation systems are essential [11,12]. In other words, the real-time information of the droplets (e.g., frequency, size, shape, speed, etc.) is needed for screening, selection, separation, and feedback control of the running droplets in microfluidic systems [13,14].

Several approaches have been used for identifying and assessment of the droplets in a running flow, which are listed in Table 1.

As listed in Table 1, stroboscope is an optical instrument that enables snapshot of cyclically moving objects. After its birth, stroboscope is widely used in the field of industry to inspect rotating wheels or vibrating strings. There are also some reports using stroboscopes to observe droplets injected from a print head [22]. Specifically, in microfluidics, stroboscopes have also been used in a centrifugal disk to observe the real-time process on spinning disks [23,24,25,26,27]. For this purpose [23], an encoder mounted on the motor shaft is needed to generate a periodic trigger signal when the shaft is at a specific angle of rotation. Based on this trigger signal, the stroboscope synchronizes with the disk, and captures progressive images of each rotation at the specific rotational angle of the disk, displaying the region of interest in the camera frame as if the images are captured while the disk is at rest. Based on this scheme, observation of valve status [25] and detection of fluorescence on spinning disk-shaped chips have been achieved [27]. Synchronized with a camera, the stroboscope has also been used in a μ-PIV system to draw flow fields in microfluidics [28,29]. Quantitative analysis in the flow system, such as enzyme kinetic analysis, was also achieved with the aid of a stroboscope [30]. To our knowledge, almost all of the existing works need a trigger signal to synchronize a stroboscope with a camera. Droplets detection and synchronization modules are therefore prerequisite components, which increase the complexity and cost of the chips and systems. Moreover, there are few reports using stroboscope in droplet-based microfluidics for real-time visualizing and modulating high-throughput droplets.

In this paper, we propose a new and facile strategy for visualizing and modulating high-throughput droplets (SVMHD) in microfluidics without a trigger or synchronizer. In the following sections, we first introduce the mechanism, setup, and method of SVMHD. Then, we verify this methodology in imaging droplets generated from a microfluidic T-junction droplet generator. Finally, as a demonstration, the SVMHD strategy has been used in a miniaturized picoinjector to enhance the reliability of the system.

## 2. Principle

Capture of moving droplets *x(t)* in microfluidics is a discrete sampling process. The sampling result *x_p_(t)* can be expressed by the original signal *x(t)* multiplying a sampling function *p(t)*. In practice, the sampling function *p(t)* is usually a periodic impulse train, which can be expressed as:

(1) $$p(t)=\sum_{k=-\infty }^{\infty }\delta \left(t-kT\right)$$

where *δ* is the Dirac delta function, *T* is the sampling period, *k* is an integer. From Equation (1), in the frequency domain, the Fourier transform of the sampling result can be expressed as [31]:

(2) $$X_{p}\;(j\times 2\pi f)=\frac{1}{T}\sum_{k=-\infty }^{\infty }X\left(j\times 2\pi \left(f-kf_{flash}\right)\right)$$

where *f_flash_ = 1/T* is the sampling frequency. Equation (2) shows that the spectrum of the sampling result is the original spectrum *f_origin_* shifted by integer multiples of the sampling frequency *f_flash_*. In other words, the frequency of the modulated sampling result (*δf*) is expressed as:

*δf* = *f_origin_* − *k* × *f_flash_* (3)

Based on this equation, we can tune the frequency of the modulated result by changing the sampling frequency *f_flash_*, and map a high frequency signal (*f_origin_*) to a lower frequency signal (*δf*).

**Figure 1:**
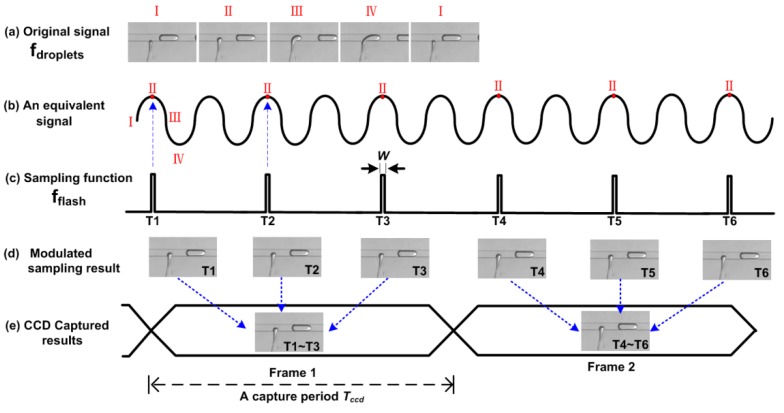
The principle of strategy for visualizing and modulating high-throughput droplets (SVMHD). T1–T6 represent the sequential specific sampling moment. (**a**) A typical T-junction droplet generator provides an original cyclical signal. (**b**) A sinusoidal wave represents the four states of the original signal in one cycle. (**c**) A periodic impulse train with duration of *W* is used as the sampling function. (**d**) The modulated sampling results show the static images of state II. (**e**) A conventional Charge Coupled Device (CCD) captures the static image in a capture period, which is a sum of modulated sampling results (as indicated by the blue arrows).

A more vivid explanation of the SVMHD principle can be found in Figure 1. Figure 1a shows a typical droplet generation cycle, the frequency of which is noted as *f_droplets_*. For convenience, we divide one droplet generation cycle into four states: I, II, III, IV. Meanwhile, we use a sinusoidal wave (its frequency is equal to *f_droplets_*) as an equivalent periodic signal to represent these four states. In one droplet generation cycle, these four states occur in sequence, following a sinusoidal wave cycle. Then, a sampling function (a periodic impulse train, *f_flash_*) is used to modulate the original signal. If its frequency satisfies *f_flash_ = f_droplets_*/*N*, where *N* is an integer (*N* = 2 in Figure 1c), the same state (state II in Figure 1) will occur at the sampling time. For this situation (*δf* = 0 in Equation (3)), the modulated result is a DC signal (Figure 1d). Hence, a conventional Charge Coupled Device (CCD) with a long capture period *T_c__cd_*, is competent to acquire the static images (Figure 1e).

In microfluidics, high-throughput droplets usually present a periodic signal. This has been proved and reported in many papers [2,11,32,33]. Hence, the phase between high-throughput droplets *f_droplets_* and the sampling function *f_flash_* can be fixed or tuned during the sampling procedure, even without external synchronization. In this way, we can remove the trigger signal and related droplet detection modules in SVMHD, which makes the strategy compatible with any droplet-based microfluidic chips.

When the sampling function *f_flash_* satisfies *δf* = 0 in Equation (3), the images of the droplets appear clear and static. If we sequentially select the frames at these sampling frequency and mark them as *f_1_*, *f_2_*, …, *f_N-1_*, *f_N_*, *f_N+1_*,…, and so on, we obtain the droplet frequency *f_droplets_* as:

*f_droplets_* = (*f_N_* × *f_N-1_*)/(*f_N_* − *f_N-1_*) = *F*(*f_N-1_*, *f_N_*) (4)

Once *f_droplets_* is figured out, the droplet velocity (*v*) in the microchannel can be calculated by:

*v* = *L* × *f_droplets_* (5)

where *L* represents the distance between two consecutive droplets in the microchannel.

Furthermore, if the sampling frequency *f_flash_* is carefully adjusted (*δf* deviates from zero a little), a resulted signal with a low frequency (*δf*) is produced. In this case, we can capture images of different states in a droplet cycle (i.e., the whole dynamic process).

## 3. Setup

**Figure 2:**
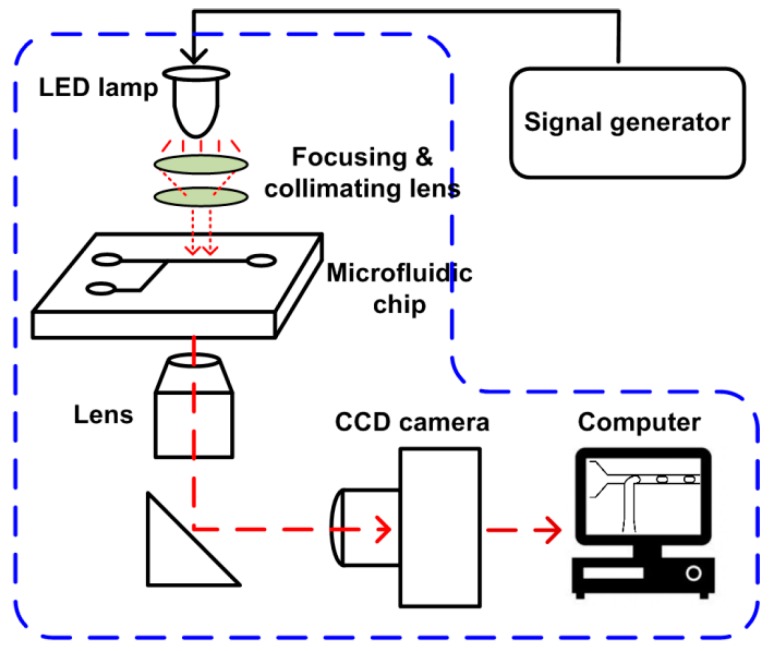
Schematic of the SVMHD setup. The blue dash box indicates the components of a regular microfluidic platform.

The setup of the SVMHD system mainly consists of three parts: an illumination part, a microfluidic chip, and an image capture part (as shown in Figure 2). In the illumination part, a LED lamp (MCWHL5-C1, Thorlabs, Newton, NJ, USA) combined with focusing lens serves as a flash light source. The LED lamp is controlled by a conventional signal generator (MHS5200A, Minghe, Zhengzhou, China) that adjusts the frequency and duty cycle of the flash light. The flash light is focused by a focusing lens, adjusted by a condenser to ensure a collimated beam be presented on the microfluidic chip. Then, the illuminated microfluidic chip image is focused by an objective lens, and then captured by a conventional CCD camera (Retiga R1, Qimaging, Surrey, BC, Canada). A computer is used to read out images from the CCD and deduce related information from these images. 

The components in the blue dash box in Figure 2 are the basic configuration of a microfluidic lab. The SVMHD only need an additional signal generator for altering a constant light source into a controllable flash light source. Therefore, the SVMHD setup is compatible with and can be easily implemented on any microfluidic labs.

## 4. Results and Discussion

### 4.1. Droplet Frequency f_droplets_

**Figure 3:**
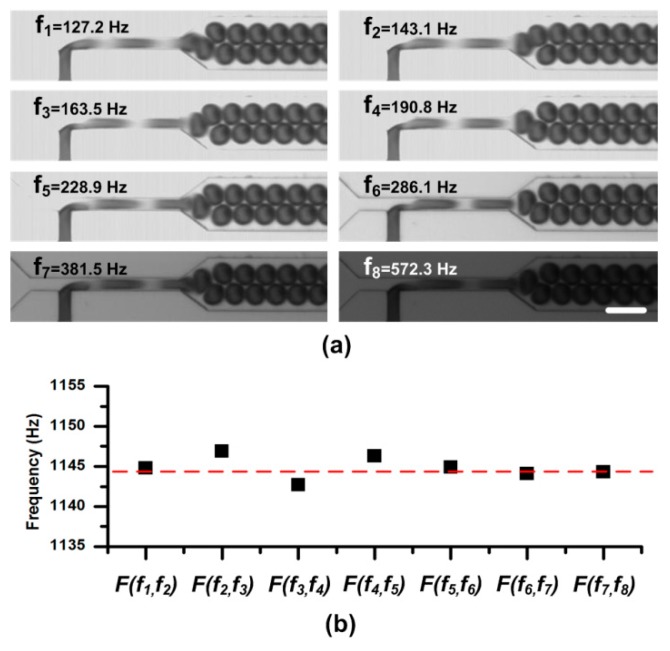
(**a**) Static droplet images at different sampling frequencies in the sweeping process. A same duty ratio of the flash light is adopted in the experiment. Therefore, the images get darker when the sampling frequency sweeps from low to high. For the CCD, a long capture period *T_c__cd_* = 100 ms (and the capture rate is 10 frames per second), is used to capture images. The scale bar is 100 μm. (**b**) The calculated frequencies from series of two consecutive sampling frequencies. The function *F(f_N-1_,f_N_)* is defined in Equation (4).

To demonstrate the SVMHD working procedure, we first illustrate the determination of the high frequency of a droplet generator. In this experiment, a T-junction microfluidic chip was used to generate high-throughput droplets (as shown in Figure 3). 

Two syringe pumps (PHD 2000, Harvard, Holliston, MA, USA) were used to pump oil and pure water into the chip. The flow rates of oil and water were kept as 4.0 μL/min and 1.4 μL/min, respectively. After the droplet generation became stable, we started to take images. Due to lack of any information about the droplet frequency, we swept the sampling frequency (i.e., the flash light frequency) *f_flash_* from low to high (e.g., 30 Hz to 0.6 kHz in this experiment), and recorded the droplet—live images with the conventional CCD. As shown in Figure 3a, we sequentially selected the sampling frequency at which the droplets were static and clear (*f_droplets_/N* in theory), and marked them as *f_1_, f_2_, …, f_k-1_*, and *f_k_*. The details about the sweeping process are available in supplementary materials Movie S1. Consequently, according to Equation (4), we obtained the droplet frequency *f_droplets_* in the microchannel. Meanwhile, as shown in Figure 3, the information about the droplet size and shape can be easily obtained from these static images. To verify the measuring results, these droplets were also imaged by a high-speed camera (OptiMOS, Q-IMAGING, Chicago, IL, USA) at the same time for calculating the droplet frequency, velocity, etc.

The frequencies calculated from two consecutive sampling frequencies were listed in Figure 3b. In the figure, the calculated droplet frequencies fell on the line 1144.9 ± 2.0 Hz which agreed well with the frequency measured from the images by the high-speed camera, 1144.5 Hz, indicated by the red line. It should be noted that, in the experiment, a long capture period (e.g., *T_c__cd_* = 100 ms in the experiment) is used to capture images of droplets, which can easily be satisfied by a general CCD.

### 4.2. Control of Motion Blur

As indicated in Equation (3), we can map a high frequency droplet signal *f_droplets_* to a low frequency signal *δf*. Hence, in theory, we can visualize high-throughput droplets without limitation in their frequency. However, during the sampling pulse duration (*W*, as indicated in Figure 1c), the moving droplet (its real velocity is *v*) is exposed and its image is captured. The moving distance (*L_blur_*) during the exposure time can be expressed as,

(6) $$L_{\mathrm{blur}}=W\times v$$

**Figure 4:**
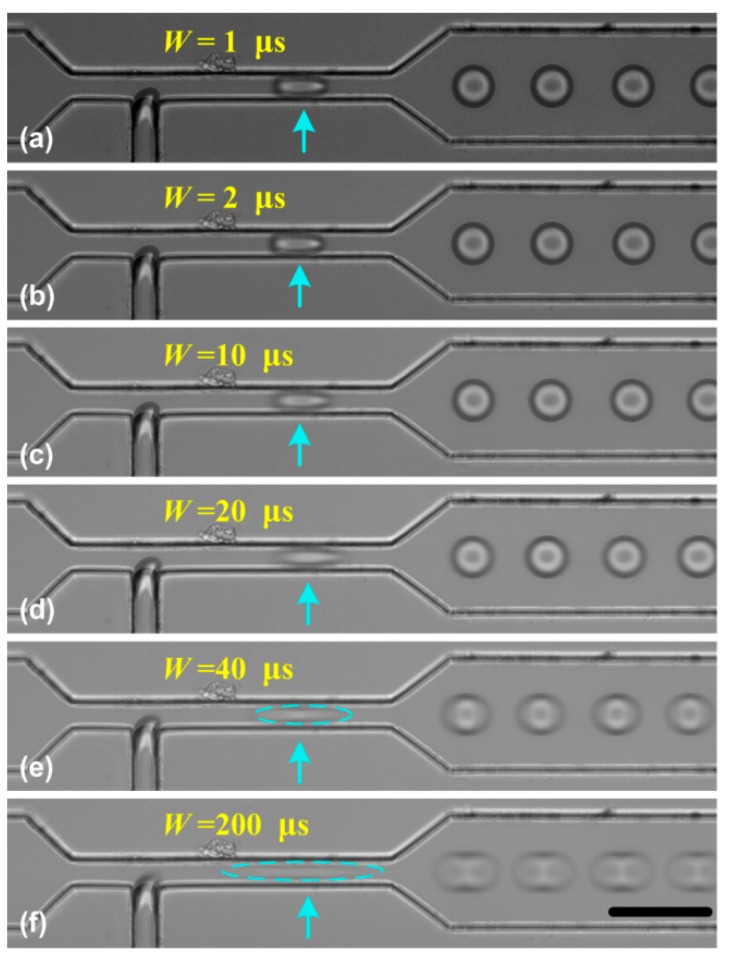
The effect of the pulse duration *W* on the droplet image. (**a**–**f**) The pulse duration stepped from 1 μs to 200 μs. In the experiment, the oil and water flow rates were respectively set as 7.0 μL/min and 1.8 μL/min. The blue arrows indicate the droplet location. The scale bar is 50 μm.

Therefore, for a faster moving droplet, *L_blur_* will become larger and the obtained image may be blurred, which may limit the usage of SVMHD. To show the effect of duration *W* on the motion blur, we performed the SVMHD in T-junction using variable pulse durations of the flash light. In the experiment, the oil and water phase flow rates were kept constant during the whole experiment. By using SVMHD, the droplet frequency *f_droplets_* was measured as 2217.7 ± 3.7 Hz. Then, we set the flash frequency *f_flash_* the same as droplet frequency, and stepped the pulse duration from 1 μs to 200 μs. The images were captured and are shown in Figure 4. 

In Figure 4a–f, as the pulse duration became longer, the blur became more obvious. Based on SVMHD, the droplet moving velocity *v* was calculated as 85.3 ± 0.2 mm/s in the wide channel. According to Equation (6), the moving distance (*L_blur_*) was estimated as 17.06 ± 0.04 μm for *W* = 0.2 ms, which is in good agreement with Figure 4f. Meanwhile, from Figure 4c–e, we find that the droplets in a narrow channel become more blurred relative to in the wide channel, although the droplet frequency is the same in both channels. As discussed above, it is the image blur that limits the scope of SVMHD. Hence, we conclude that the scope of SVMHD is limited by the droplet real velocity and the achievable pulse duration, rather than the droplet frequency. 

In droplet-based microfluidics, although the frequencies of high-throughput droplets can be up to several tens of kilohertz [34,35], their velocities are usually less than 1 m/s. For a conventional LED lamp, the shortest pulse duration is within several nanoseconds. Therefore, the motion blur can be limited to a few microns, which can be ignored when compared with the size of droplet (usually several or several tens microns). There are also brighter and faster response light sources (such as Xenon flash lamp [27], super-bright light emitting diodes [36]), which helps to ensure enough exposure dose during short pulses. Therefore, it is concluded that the SVMHD can be used in any high-throughput droplet-based microfluidics without limitations.

### 4.3. Tests in a Miniaturized Picoinjector System

In droplet-based microfluidics, adding reagents into droplets is one of the most important functions. We have developed a new picoinjector for precise control of the volume of the reagent injected into droplets at kilohertz rates [37]. To enhance its reliability, we used SVMHD to capture the dosage information, which supplied a feedback signal to dose the injection volume in a closed-loop control system.

#### 4.3.1. Visualization of the Injection Process

The SVMHD was firstly used to visualize the whole injecting process in the picoinjector. In the experiment, using the method described above, the droplet frequency *f_droplets_* was measured as 247.2 Hz. Then, the flash frequency *f_flash_* was set to 246.2Hz. Therefore, a low frequency signal (*δf* = 1 Hz) was generated corresponding to the injection process in a droplet injection cycle.

**Figure 5:**
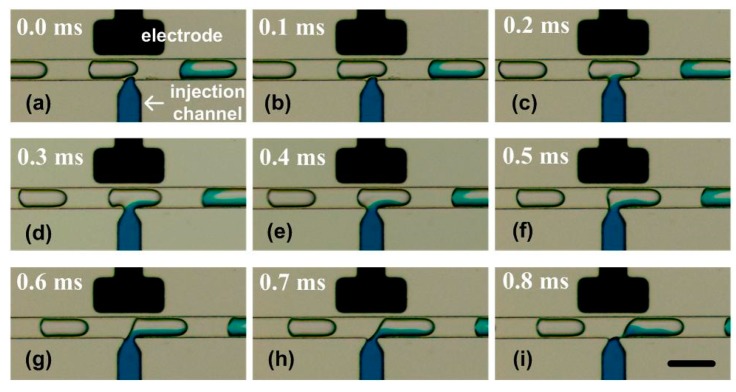
The whole injecting process in the picoinjector visualized by SVMHD. (**a**–**i**) The injecting process in a picoinjector. A colour fluid was injected into the droplets moving from left to right by the nozzle. The droplet frequency (*f_droplets_*) was tested as 247.2 Hz, and the flash frequency (*f_flash_*) was set as 246.2 Hz. In the experiment, the capture period (*T_c__cd_*) was 100 ms and the capture rate was 10 frames per second. The scale bar is 100 μm.

The injection process was clearly visualized in the captured consecutive images. Prior to the injection, a constant voltage (e.g., 10 volts) was applied to the electrodes, and the adding reagent was confined behind the nozzle. When a droplet (with a volume of *V_ini_*) moved along the microchannel and passed the injection nozzle (Figure 5a), the electric field at the oil/water interface was enhanced as the dielectric constant of the water was much higher than that of the oil. The interface ruptured due to electrically induced thin-film instability [37] (Figure 5b). Thus, the reagent from the injection channel entered the droplet (Figure 5c). Next, the reagent injection process continued (Figure 5c–f) until the rear end of the droplet approached the edge of the nozzle (Figure 5g, the duration is represented as *t*). Then, the neck became longer and thinner (Figure 5h) and finally pinched off (Figure 5i). A droplet with a volume of *V_total_* was ultimately generated in the main channel. After the separation, the tip of the adding reagent stream rapidly retracted to the nozzle. The system was stabilized and then ready for the next injection cycle.

The injection volume (*V_dose_*) can be calculated from the changes between the droplet volume before and after the injection process. In the calculation, a droplet is modeled as a cylinder with two caps, and its volume is determined by the distance between its leading and trailing interfaces.

#### 4.3.2. Enhancing System Reliability with SVMHD

**Figure 6:**
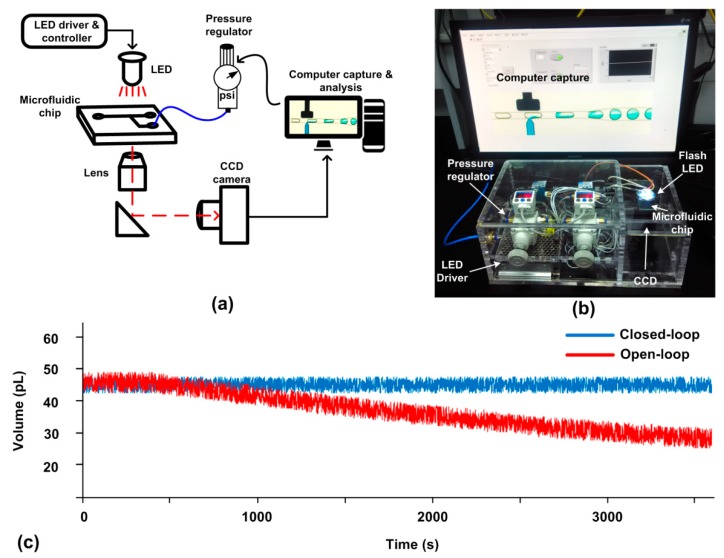
(**a**) Schematic of the components in a picoinjector system. (**b**) An image of the real miniaturized picoinjector system including a LED lamp, a signal generator, a picoinjector microfluidic chip, a CCD camera, a computer and a pressure regulator (Type 2000, Marsh Bellofram, Newell, WV, USA). (**c**) The droplet volume results calculated by the computer in a 60 min duration. In the experiment, the capture period (*T_c__cd_*) was 100 ms and the capture rate was set as 4 frames per second. The red line represents the result of the open-loop picoinjector while the blue line represents the result of the closed-loop picoinjector.

The above picoinjector [37] is an open-loop system without control of the injection dosage. To achieve a uniform dosage and enhance the reliability of the picoinjector, we used SVMHD to capture the dosage, and supplied a feedback signal to the picoinjector. The schematic of a miniaturized closed-loop control system is shown in Figure 6a.

As shown in Figure 6a, the fast-moving droplet images were captured by CCD and a computer using SVMHD. At the same time, the computer analyzed the images and calculated the injection dosage. According to the error (real dosage relative to the aimed dosage, *ΔV*), the computer adjusted the required injection pressure (*ΔP*) to achieve the aimed dosage. In such a way, the injection dosage was adjusted in a closed-loop control system. 

We tested the performance of the closed-loop miniaturized picoinjector. For comparison, the open-loop was also tested at the same condition except for the feedback control. Their results were listed in Figure 6c. At the beginning, the dosages of both systems were set as 45 pL. During the following 60 min, the dosage of the closed-loop picoinjector was almost kept constant, 45 ± 2.4 pL. In contrast, the dosage of the open-loop picoinjector was drifting gradually to 36 ± 3.6 pL after 60 min. The pressure drift or channel dimension changing in the real system might be the reasons for dosage drift in the open-loop picoinjector. This result manifests that with the aid of the feedback control offered by SVMHD, the reliability of the picoinjector is significantly enhanced.

Another big concern is the application scope of SVMHD. In droplet-based microfluidics, many biochemical reactions are photosensitive (such as, photo curing [38], fluorescence quenching [39], and so on [40]). To visualize droplets in this case, we need to carefully control the spectra of flash light (using suitable optical filters or selecting LEDs with specific spectra) in SVMHD to avoid triggering unwanted reactions in droplets.

## 5. Conclusions

To our knowledge, this is the first report about a facile strategy for visualizing and modulating droplets in microfluidics without an additional trigger signal. By taking advantage of highly periodicity of high-throughput droplets, the SVMHD provides an imaging solution alternative to a high-speed camera with many inherent characteristics: (1) Low-cost. In SVMHD, instead of using a high-speed camera (expensive and bulky), a conventional CCD can be used to visualize and capture high-throughput droplets in microfluidics. Furthermore, when compared with the professional stroboscope, the droplet detection and synchronization modules are not necessary. This is particularly appealing for constructing a miniaturized system. (2) Compatible. In SVMHD, the key component is a controllable flash light, which can be easily achieved by a signal generator and a LED and integrated into a microscope or any other homemade optical system. (3) Powerful. Using SVMHD, information of the high throughput droplets, such as frequency, shape, size, and velocity can be obtained. Furthermore, not only a static image, but also a dynamic process can be captured. (4) Real-time. SVMHD can map a high frequency signal to a lower frequency signal. It is convenient to handle this low frequency signal in real-time. Therefore, SVMHD can, not only monitor the process in microfluidics, but also supply a feedback signal to modulate microfluidic systems.

In all, due to these advantages, we expect this strategy to be easily implemented in miniaturized microfluidic systems or microfluidic laboratories, and supply low-cost and functional equipment for droplet-based microfluidics.

## Figures and Tables

**Table 1:** Different approaches identifying moving droplets in microfluidics.

Approaches	Advantages	Disadvantages
High Speed Camera [15]	Can capture the size, shape of droplets with a high frame rate (≥1000 fps)	Expensive and bulky, and will produce a huge amount of data.
CMOS/CCD sensors [16,17,18,19]	Can capture the size, shape of droplets. Low-cost.	A lower capturing frame rate (≤100 fps). Be not competent to observe high throughput droplets.
Photo detector [20]	Low-cost and easy setup	Be limited for droplets recognizing and counting.
Moving shot [21]	Can capture the size, shape of droplets.	Needs to know the droplet moving velocity firstly. Costly.
Stroboscope	Can capture the size, shape of droplets in microfluidic devices or on spinning disks. Be competent to observe high throughput droplets. Low-cost.	Needs synchronization signals. Few reports about using strobe in microfluidics.

## Supplementary Materials

The following are available online at https://www.mdpi.com/2072-666X/10/5/291/s1, Movie S1: The SVMHD sweeping process for deciding the droplet frequency. Movie S2: A side-by-side video comparison of SVMHD with a high-speed camera. Note, in Movie S2, there is spatial distortion of droplet images that captured by the high-speed camera due to the Rolling Shutter readout mode. Figure S1: Comparison of open and close loops of picoinjector in working principle.
