# Supplementary material for: A Facile Strategy for Visualizing and Modulating Droplet-Based Microfluidics
Source: Micromachines (Basel). 2019 Apr 29;10(5):291. doi: 10.3390/mi10050291 (PMC6562635; doi:10.3390/mi10050291)
Supplement: Supplementary file 1 [file micromachines-10-00291-s001.zip › supplementary-for publish/Supplementary Material.docx]

Supplementary Materials: A Facile Strategy for Visualizing and Modulating Droplet-Based Microfluidics


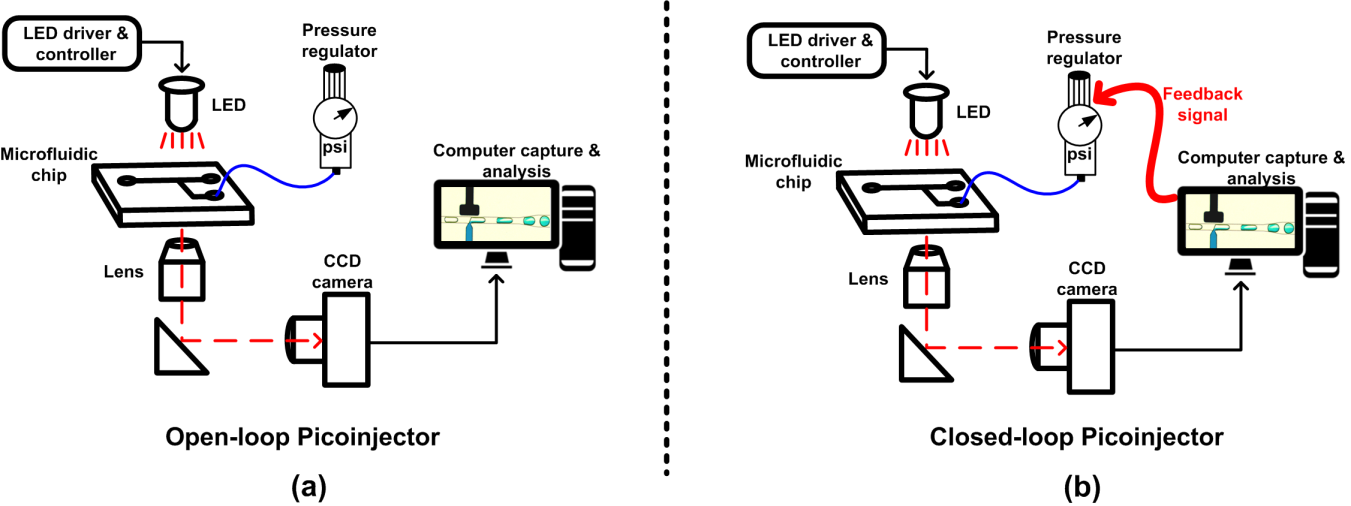


**Figure S1.** Comparison of open and close loops of picoinjector in working principle.

The main difference is that open-loop picoinjector has no feedback signal. On the contrary, in closed-loop picoinjector, the droplet images are captured by a conventional Charge Coupled Device (CCD) and a computer using strategy for visualizing and modulating high-throughput droplets (SVMHD). Then, according to the droplet images, the computer sends a feedback signal and adjusts the injection pressure.

| 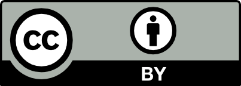 | © 2019 by the authors. Submitted for possible open access publication under the terms and conditions of the Creative Commons Attribution (CC BY) license (http://creativecommons.org/licenses/by/4.0/). |
| --- | --- |
